# Supplementary material for: Droplets in underlying chemical communication recreate cell interaction behaviors
Source: Nat Commun. 2022 Jun 1;13:3047. doi: 10.1038/s41467-022-30834-2 (PMC9160030; doi:10.1038/s41467-022-30834-2)
Supplement: Supplementary file 1 — Supplementary Information [file 41467_2022_30834_MOESM1_ESM.pdf]

Supplementary Information

Droplets in Underlying Chemical Communication Recreate Cell Interaction Behaviors

Agustin D. Pizarro,[1] *Claudio L. A. Berli*[2] *Galo J. A. A. Soler-Illia,*[1]

and, Martín G. Bellino [3,*]

1- Instituto de Nanosistemas, UNSAM-CONICET, Av. 25 de Mayo 1021, 1650 San Martín, Buenos Aires, Argentina.

2- INTEC (Universidad Nacional del Litoral-CONICET) Predio CCT CONICET Santa Fe, RN 168, 3000 Santa Fe, Argentina.

3- Instituto de Nanociencia y Nanotecnología (CNEA-CONICET), Av. Gral. Paz 1499, San Martín, Buenos Aires, Argentina. email: *mbellino@cnea.gov.ar*

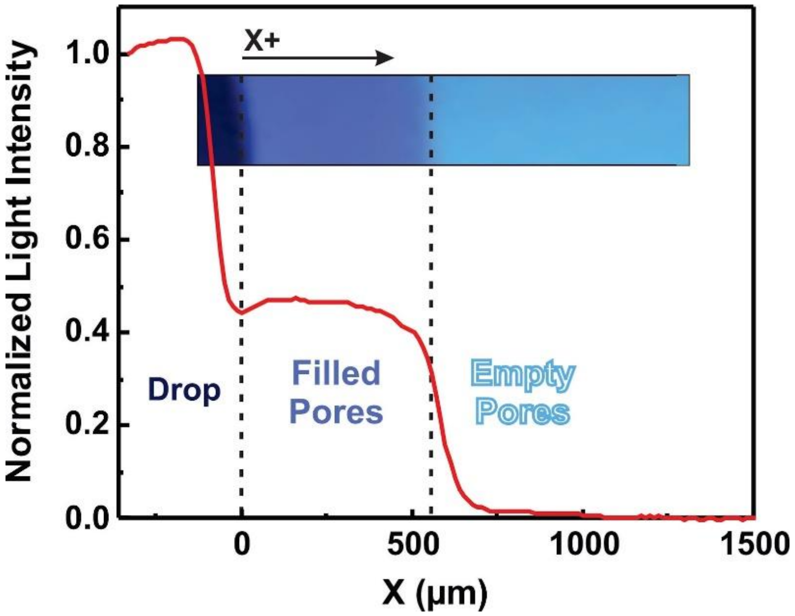

Supplementary Fig. 1: Spatial pore filling on the nanoporous film mapped by light intensity profile. Normalized light intensity profile from the drop border up to a zone far-off the droplet after the steady wetted annulus was self-generated into the nanopore network around a 1 wt% KI drop deposited on a nanoporous titania thin film (see Supplementary Fig. 2 for film characterization details) (T = 25 °C and 45% RH). The light intensity reflected by the nanoporous film can be employed an indicator of pore filling.[1] The reflected light intensity is considered to be proportional to the amount of fluid in the films, which is based on the linear approximation for the effective refractive index: $n_{eff} = \sum_i n_i f_i$, where n_i and f_i are the refractive index and volume fraction of each component (nanoporous titania, liquid, and air).[2] This approximation implies that the pore filling- light reflectance relationship is rather linear, as it was recently suggested.[3] A recorded image was processed in MATLAB to extract a reflected light intensity profile as a measure of the pore filling profile. The information considered was that from the green channel (RGB system). Light intensity was considered at different positions along the radial direction, in an elongated domain that includes the wet annulus and the region away from the drop. The inset presents an image capture showing the region of analysis and the reference system.

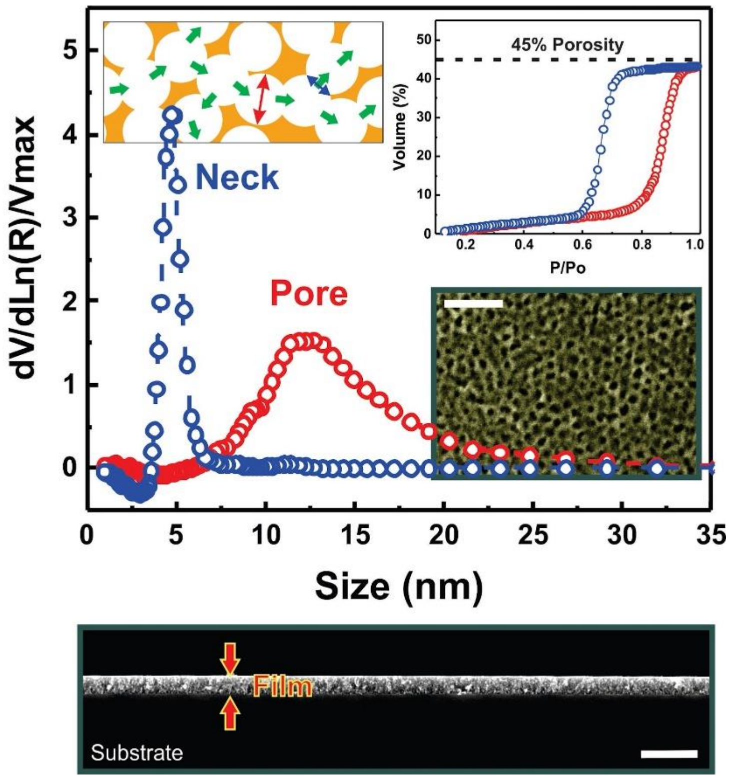

Supplementary Fig. 2: Structural characteristics of the nanoporous film used in this study. Typical pore and neck size distribution (Inset: water adsorption-desorption isotherms at 298 K) obtained from environmental ellipsometric porosimetry (EEP) analysis. The EEP technique allows assessing the porosity of the nanoporous films.[4] The water adsorption–desorption measurement resulted in type IV isotherm with a H1-like hysteresis loop (according to IUPAC classification), characteristic of the formation of open channel type pores.[5] Delayed closure of the hysteresis loop is attributed to pore constrictions (necks) along the main structural mesoporosity[6] (see simplified scheme where green arrows indicate the arbitrary channel paths and red and blue double-head arrows point to the pore and neck sizes, respectively). Film thickness and the real component of the refractive index were obtained from the ellipsometric parameters $\psi(\lambda)$ and $\Delta(\lambda)$ in the 400-800 nm spectral range.[7] In this range, no light absorption is observed, thus only the real part of the refractive index (n) is considered. The model used for fitting consisted of an oxide film supported on a silicon substrate. The substrate and film refractive index (n) were described according to a modified Cauchy equation: $n(\lambda) = A + B/\lambda^2$, where A and B parameters were extracted from the fit of $\Psi(\lambda)$ and $\Delta(\lambda)$. Water adsorption isotherms were measured by EEP according to the protocols developed in ref [8]. The samples to be analyzer were placed under a continuous flux of nitrogen containing variable water

vapor quantities (fixed partial water pressures). Film thickness and refractive index values were obtained at each relative pressure value, P/P_0, where P is the actual vapor pressure measured by a humidity probe and P_0 being the saturation water vapor at 298 K. Relative pressures were varied from 0 to 1 to obtain the whole adsorption curve. Film pore volume was evaluated by adjusting the refractive index at λ=633 nm with a two-medium Bruggeman effective medium approximation (BEMA), in $P=0$ (dry nitrogen flux, nitrogen/oxide) and $P=P_0$ (water saturated nitrogen flux, air/oxide). Water uptake at intermediate pressures and pore size distributions were obtained using WinElli 2 software (Sopra Inc.), which transforms the refractive index variation with P/P_0 into filled pore volume by using a three-medium (air, water, oxide) BEMA. Pore size distributions were obtained from the adsorption and desorption curves of the water EEP isotherm using a model based on an adapted Kelvin's equation.[8] Pore diameter is deduced from the adsorption curve and the dimension at the desorption is representative to the neck diameter in the structural porosity.

The inset top-view SEM image in the lower right corner that shows the nanopores (scale bar = 50 nm) and the cross-sectional view (bottom frame) that reveals the thin nature of the nanoporous film (scale bar = 500 nm) were added in order to complete the structural characterization.

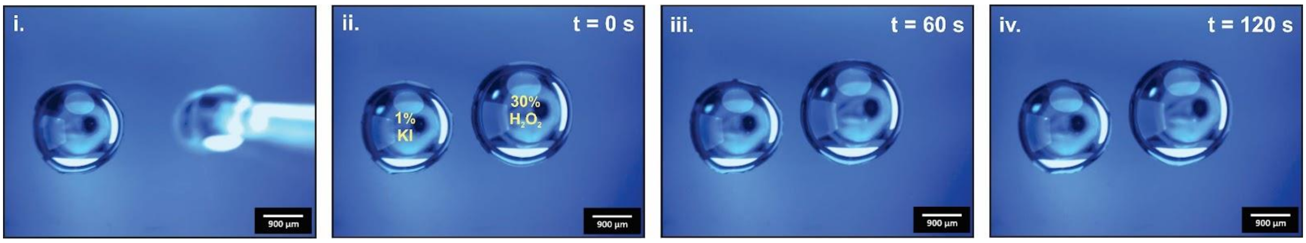

Supplementary Fig. 3: Silicon Substrate Control. Time-lapse snapshot sequence showing a 30 wt% H_2O_2 drop placed in the vicinity of a 1 wt% KI droplet on the silicon substrate (in the absence of the nanoporous film coating). Note that no annulus were generated around the droplets on both surfaces. No droplet response was observed.

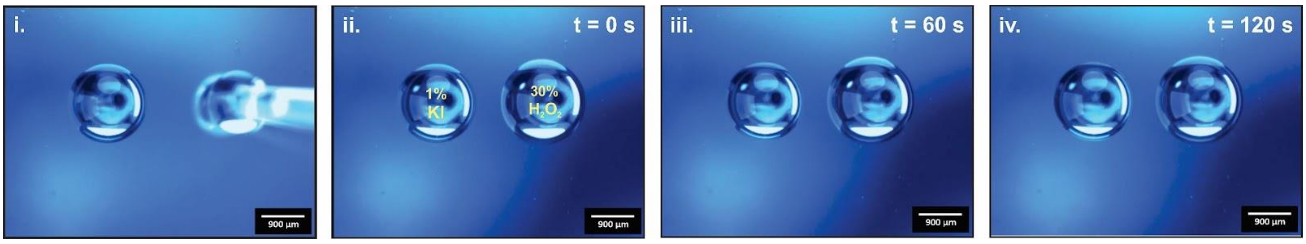

Supplementary Fig. 4: Dense Titania Film Control. Time-lapse snapshot sequence showing a 30 wt% H_2O_2 drop placed in the vicinity of a 1% KI droplet on a dense titania thin film (in the absence of template). Note that no annulus were generated around the droplets on both surfaces. No droplet response was observed.

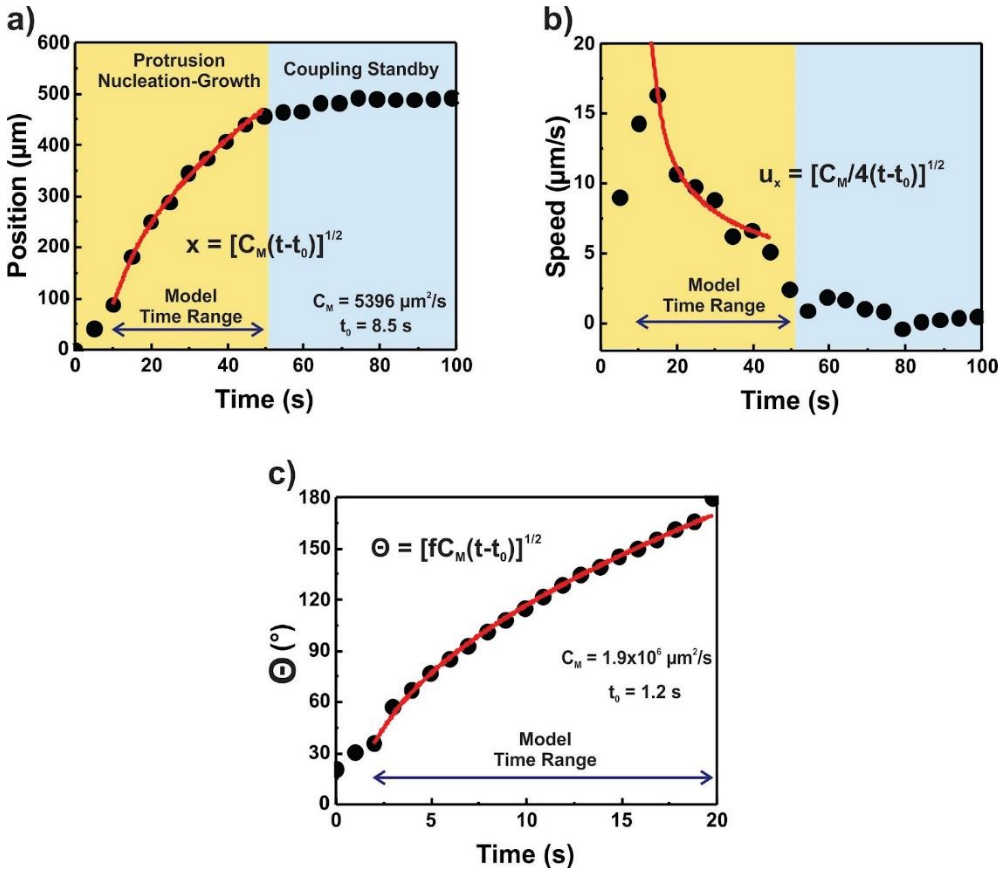

Supplementary Fig. 5: Model Prediction. Analysis of experimental data according to the square-root-of-time kinematics equation predicted by the model for protrusion advance (see equation insets, were c_{M} is defined as the Marangoni flow coefficient and t_0 is associated to the time required to reach the Marangoni flow regime). Symbols are experimental data and lines are the prediction of the equation for temporal dependence of: (**a**) position and (**b**) velocity of the advancing protrusion reported in Fig. 3 and (**c**) rotational angle along the circular path reported in Fig. 6. In the latter, the model equation includes the factor $\mathrm{f} = (180/\pi r)^2$ that converts angular units (°) to arc length units (µm), where $r \approx 2$ mm is the radius of the circular paths (see Fig. 6b in the main text).

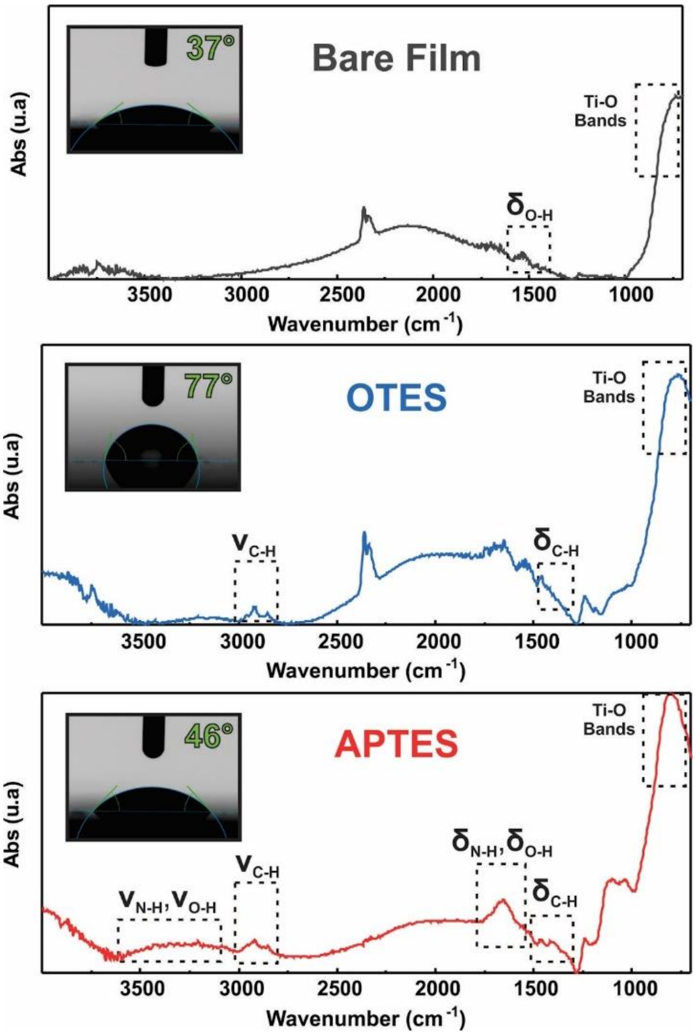

Supplementary Fig. 6: Surface modification details. Fourier transform infrared (FTIR) characterization on octyltriethoxysilane (OTES) and (3-aminopropyl)triethoxysilane (APTES) modified nanoporous titania films, using bare titania film for comparison. FTIR spectra of surface-modified TiO_2 nanoporous films show characteristic bands of organic molecules attached to the titania surface. The contact angle insets illustrate the wettability changes from surface modification. FTIR spectra were recorded on a FTIR Thermo Nicolet iN-10 Ultrafast MX – ATR Mode CA Microscope. Contact Angle measurements were performed on a Krüss DSA25 Expert Analyzer.

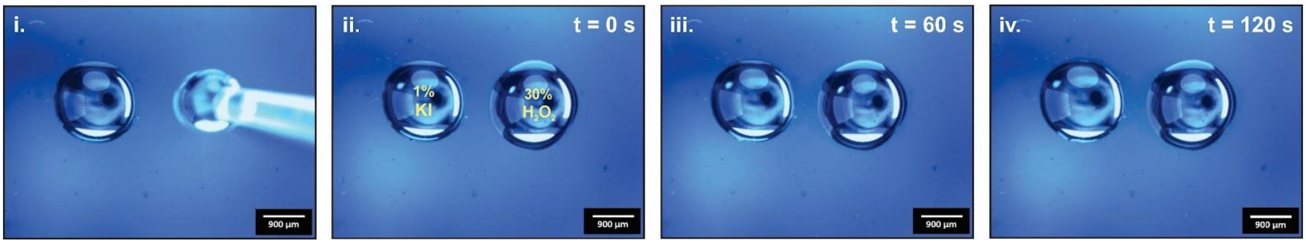

Supplementary Fig. 7: OTES-Functionalized Film. Time-lapse snapshot sequence showing a 30 wt% H_2O_2 drop placed in the vicinity of a 1wt% KI droplet on an OTES-modified nanoporous film. A practically negligible annulus was generated in the KI drop periphery. No droplet response was observed.

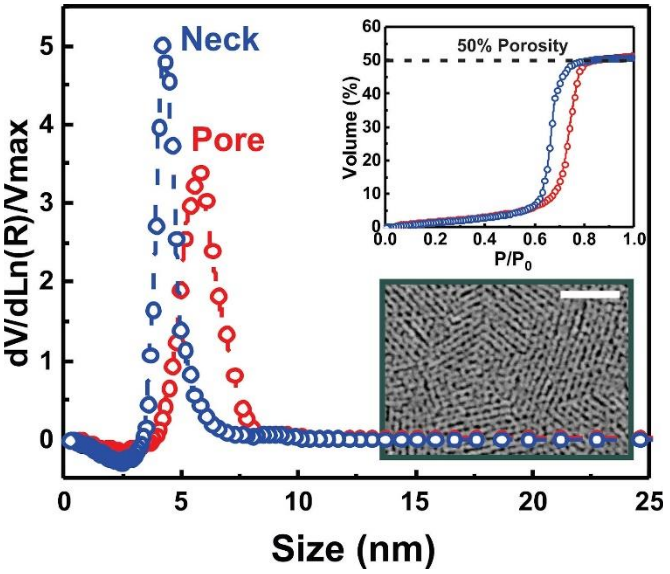

Supplementary Fig. 8: Small-Pores Film Characterization. Typical pore and neck size distribution obtained from environmental ellipsometric porosimetry analysis of the nanoporous thin film with smaller pores. Insets: water adsorption-desorption isotherms at 298 K. SEM imagine was added in order to complete the structural characterization (scale bar = 100 nm). The nanoporous coatings produced by the fast-firing thermal treatment result 180 nm thick, 50% porous and exhibit pore and neck sizes of 6 and 4.1 nm, respectively. The minor hysteresis observed in the water adsorption-desorption isotherm with respect to the above film is associated to a smaller difference between the neck and the pore diameters (presenting domains of conduit-like aligned pores).

Supplementary Table 1: Summarized structural characteristics obtained from ellipsoporosimetry of the nanoporous titania films.

	Pore Diameter (nm)	Neck Diameter (nm)	Thickness (nm)	Pore Volume (%)
Large-Pores Film	12	4.5	180	45
Small-Pores Film	6	4.1	180	50

References

[1] Urteaga, R., Mercuri, M., Gimenez, R., Bellino, M. G. & Berli, C. L. Spontaneous water adsorption-desorption oscillations in mesoporous thin films. *Journal of Colloid and Interface Science*, **537**, 407-413 (2019).

[2] Urteaga, R. & Berli, C.L. In Nanoporous Alumina: Fabrication Structure, Properties and Applications, in: D. Losic, A. Santos (Eds.), Springer Series in Materials Science, Springer International Publishing, pp. 249–268. Chapter 8 (2015).

[3] Vincent, O., Marguet, B. & Stroock, A.D. Imbibition triggered by capillary condensation in nanopores. *Langmuir* **33**, 1655–1661 (2017).

[4] Baklanov, M. R., Mogilnikov, K. P., Polovinkin, V. G. & Dultsev, F. N. Determination of pore size distribution in thin films by ellipsometric porosimetry. *Journal of Vacuum Science & Technology B: Microelectronics and Nanometer Structures Processing, Measurement, and Phenomena,* **18**, 1385-1391 (2000).

[5] Sing, K. S. Reporting physisorption data for gas/solid systems with special reference to the determination of surface area and porosity (Recommendations 1984). *Pure and Applied Chemistry*, **57**, 603-619 (1985).

[6] Zhang, P., Wang, L., Yang, S., Schott, J. A., Liu, X., Mahurin, S. M., Huang, C., Zhang, Y., Fulvio, P. F., Chisholm. M. F. & Dai. S*. Solid*-state synthesis of ordered mesoporous carbon catalysts via a mechanochemical assembly through coordination cross-linking. *Nature Communication* **8,** 15020 (2017)

[7] Tompkins, H. G. & McGahan, W. A Spectroscopic Ellipsometry and Reflectometry: A User's Guide; John Wiley & Sons: New York, 1999.

[8] Boissiere, C., Grosso, D., Lepoutre, S., Nicole, L., Bruneau, A. B. & Sanchez, C. Porosity and mechanical properties of mesoporous thin films assessed by environmental ellipsometric porosimetry. *Langmuir* **21**, 12362-12371 (2005).
