# Supplementary material for: Droplets in underlying chemical communication recreate cell interaction behaviors
Source: Nat Commun. 2022 Jun 1;13:3047. doi: 10.1038/s41467-022-30834-2 (PMC9160030; doi:10.1038/s41467-022-30834-2)
Supplement: Supplementary file 3 — Description of Additional Supplementary Files [file 41467_2022_30834_MOESM3_ESM.pdf]

Description of Additional Supplementary Files

File Name: Supplementary Movie 1
Description: Droplet Bridging Dynamics.

File Name: Supplementary Movie 2
Description: Water / KI Control.

File Name: Supplementary Movie 3
Description: H_2O_2 / KCl Control.

File Name: Supplementary Movie 4
Description: Droplet Engulfing Dynamics.

File Name: Supplementary Movie 5
Description: 0.5 wt% KI / 30 wt% H_2O_2.

File Name: Supplementary Movie 6
Description: 0.1 wt% KI / 30 wt% H_2O_2.

File Name: Supplementary Movie 7
Description: APTES Functionalization.

File Name: Supplementary Movie 8
Description: Droplet Dynamics on a Small-Pore Surface.
